# Evaluation of the toxicity and efficacy of a multi-target polymer-drug nano-polyplex in SH-SY5Y cells and *Drosophila* model of tauopathy

**DOI:** 10.1038/s41598-025-22924-0

**Published:** 2025-11-11

**Authors:** Nuruddin Mahadik, Sri Nithya Paruchuri, Rohina Arif, Amanda S. Coutts, Gemma A. Barron, Paul Kong Thoo Lin, Shreyasi Chatterjee, Colin J. Thompson

**Affiliations:** 1 School of Pharmacy, Applied Sciences and Public Health, Robert Gordon University, Aberdeen, AB10 7GJ UK; 2 Department of Science and Technology, Nottingham Trent University, Nottingham, NG11 8NS UK

**Keywords:** Polymer-drug conjugates, Nano-polyplex, Tau, *Drosophila melanogaster*, Neurodegenerative diseases, Alzheimer's disease, Drug delivery

## Abstract

Hyperphosphorylated tau contributes to synaptic damage and neuronal dysfunction in neurodegenerative diseases such as Alzheimer’s disease (AD), making it a key therapeutic target. This study evaluated the toxicity and therapeutic potential of a novel polymer-drug nano-polyplex, N5NM15, and polyacrylic acid (PAA) in *Drosophila* tauopathy models and undifferentiated human SH-SY5Y cells. Cellular uptake was demonstrated by N5NM15, and SH-SY5Y cell viability was significantly enhanced (45%, *p* ≤ 0.0001) under okadaic acid-induced stress, and total tau levels were reduced (1.43-fold, *p* ≤ 0.01). In comparison, PAA had a modest effect on decreasing tau phosphorylation (1.3-fold) at the pSer202/pThr205 site. Toxicity studies in *Drosophila* revealed that N5NM15 (3.5:1 and 44:12.5 µg/mL) and PAA (44 µg/mL) were toxic to adult flies expressing the eye-specific driver (*GMR*-GAL4) but were well-tolerated in flies overexpressing the pan-neuronal driver *ELAV*-GAL4. Furthermore, treatment with N5NM15 and PAA did not improve the ommatidial arrangement, eye bristle count, or eye length in tauopathy models. Climbing and survival assays indicated a potential mild protective effect at a lower concentration (3.5:1 µg/mL) at the early stage of the disease, but at a higher dose (44:12.5 µg/mL) was significantly toxic, in both wild-type (*p* ≤ 0.0001) and tauopathy models (*p* < 0.05). These findings highlight the need for N5NM15 and PAA dose optimisation and reformulation with non-toxic buffers to enhance therapeutic potential while minimising adverse effects in normal and *Drosophila* tauopathy models for AD treatment.

**Supplementary Information:**

The online version contains supplementary material available at 10.1038/s41598-025-22924-0.

## Introduction

Alzheimer’s disease (AD), the most prevalent type of dementia, is a progressive, neurodegenerative disease (NDs)^1^. Over 27 million people worldwide, or nearly 70% of all dementia cases, are affected by AD, and most of these cases are sporadic, with age being the primary risk factor^2,3^. The World Health Organisation (WHO) reported that AD and various other forms of dementia were ranked seventh amongst the leading causes of mortality in 2021^4^. Although the exact cause of AD remains unclear, several pathological abnormalities have been identified within the central nervous system (CNS) that contribute to neuronal death^5^. Among these, amyloid-beta (Aβ) plaques and neurofibrillary tangles (NFTs) of hyper-phosphorylated and aggregated tau protein are considered major hallmarks in disease progression^5^. However, Aβ plaques can form years or decades before dementia onset but have minimal effects on cognition and brain health, whereas tau NFTs are strongly associated with neurodegeneration and cognitive decline^6^. Tau tangles initially cause mild cognitive changes in the medial temporal lobes but spread to the isocortex, leading to significant impairment^6^.

Tau is a highly conserved protein with six isoforms in the human brain, crucial for microtubule assembly and axonal stability^7^. In NDs such as AD and frontotemporal dementia (FTD), tau forms aggregate and paired helical filaments, contributing to synaptic dysfunction and neuronal death^7^. Targeting tau has recently gained attention as a potential approach for treating AD, with several small molecules advancing to clinical trials^8^. These molecules modulate tau expression, aggregation, post-translational modifications, degradation, and microtubule stabilisation^8^. For instance, hydromethylthionine mesylate (HMTM), a stabilised form of methylthioninium developed by TauRX^9^, selectively inhibits tau aggregation in cell-free systems and cellular and murine tauopathy models. While two independent phase III trials showed HMTM to be safe and effective as a monotherapy for mild-to-moderate AD, its efficacy diminished when used alongside symptomatic treatments like donepezil, rivastigmine, or memantine^9^. Given the failures of many single-target molecules in clinical trials, the development of multi-target therapies has emerged as a promising strategy for enhancing efficacy ^8,10^. These approaches could mark a transformative breakthrough in AD treatment. The main limitations of multi-target small molecule compounds are their poor aqueous solubility and limited site-specific uptake, resulting in low bioavailability^11,12^.

**Fig. 1:**
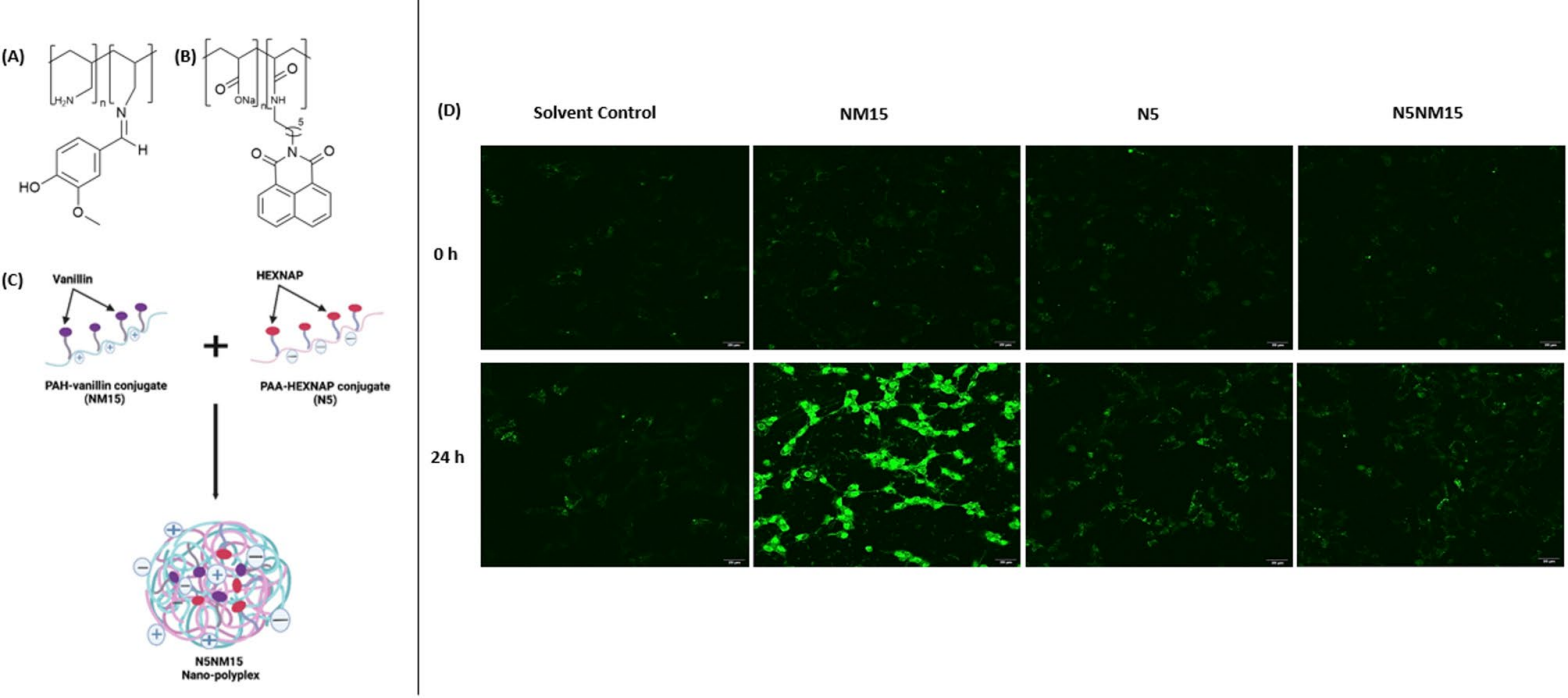
Illustrates chemical structures of (**A**) NM15 and (**B**) N5. (**C**) Schematic representation of the formation of an N5NM15 nano-polyplex between N5 and NM15 in solution. (**D**) Confocal fluorescence microscopy images depicting cellular uptake of NM15, N5, and N5NM15 nano-polyplex in undifferentiated SH-SY5Y cells incubated in serum-free DMEM media at 37 °C, with 5% CO_2._ Images taken after 0 h and 24 h (n = 3, Scale bar = 20 µm).

Nanotechnology-based drug delivery systems, such as polymeric nanoparticles, offer a promising solution to overcome these limitations^13^. Previous studies^14,15^ by our group developed novel water-soluble polymer-drug conjugates (PDCs), polyallylamine hydrochloride-vanillin (NM15), and polyacrylic acid-naphthalimidohexylamine (N5) (Fig. 1A and B). NM15 demonstrated significantly enhanced antioxidant activity (IC_50_ of 90.85 μg/mL in the DPPH assay,* p* ≤ 0.0001), while N5 exhibited significantly enhanced cholinesterase inhibitory activity (IC_50_ of 0.56 μg/mL for AChE and 0.91 μg/mL for BuChE in the Ellman’s assay,* p* ≤ 0.0001). These conjugates were then combined to formulate the N5NM15 nano-polyplex (Fig. 1C), which formed uniform nanoparticles with an average size of 30.5 ± 7.9 nm. The N5NM15 nano-polyplex demonstrated potent antioxidant activity (7494 µmolTE/1g in the ORAC assay, * p* ≤ 0.001), cholinesterase inhibition (IC_50_ of 0.54 μg/mL for AChE (*p* ≤ 0.0001) and 2.26 μg/mL for BuChE (*p* ≤ 0.01) in the Ellman’s assay), inhibition of BuChE activity in undifferentiated SH-SY5Y cells (*p* ≤ 0.01), and protective effects against oxidative stress (45%, *p*≤ 0.0001) in undifferentiated SH-SY5Y cells. N5NM15 also showed anti-inflammatory activity (> 20%,* p* ≤ 0.05) and provided substantial protection against oxidative stress in BV-2 cells (>15%). Furthermore, in a cell-free assay, N5NM15 significantly inhibited Aβ aggregation (> 10%, *p* ≤ 0.01), a key pathological hallmark of AD, suggesting their potential to modulate key disease pathways. Notably, polyacrylic acid (PAA) alone demonstrated significant protective (> 35%, *p* ≤ 0.0001) and anti-inflammatory effects (> 75%, *p* ≤ 0.0001) in both cell lines in vitro and inhibited Aβ aggregation (> 20%, *p* ≤ 0.001). These findings highlight the potential of N5NM15 and PAA as promising therapeutic candidates for neurodegenerative diseases. However, the strategy of developing PDC-based nano-polyplexes remains largely unexplored as a multi-target treatment for NDs and other diseases.

Therefore, this research was focused on evaluating the toxicity and efficacy of N5NM15 nano-polyplex and its individual polymer component, PAA, in undifferentiated human SH-SY5Y cells and the *Drosophila* model of tauopathy. The objectives were to: (i) determine the cell uptake of NM15, N5, and N5NM15 nano-polyplex in undifferentiated SH-SY5Y cells, (ii) assess the neuroprotective effect of N5NM15 and PAA against okadaic acid (OA)-induced neurotoxicity in undifferentiated SH-SY5Y, (iii) determine tau protein expression in undifferentiated SH-SY5Y cells, with and without the presence of OA, (iv) assess the toxicity of N5NM15 and PAA in *GMR*-GAL4 and *ELAV*-GAL4 *Drosophila* models, (v) investigate phenotypic changes in *GMR*-GAL4, *GMR*- hTau^2N4R^ and *GMR*- hTau^2N4R+GSK3β^ *Drosophila* models following treatment with N5NM15 and PAA, and (vi) evaluate behavioural and survival outcomes in *Elav*-hTau^2N4R^ and *Elav*-Oregon-R (WT) *Drosophila* models following treatment with N5NM15.

To the best of our knowledge, the present study is among the first to explore the in vivo application of PDC-based nano-polyplexes in a *Drosophila* model of tauopathy. This work provides preliminary insights into the toxicity profile and therapeutic potential of PDCs in the context of impacting tau levels. Our findings support the use of *Drosophila* as a valuable model for early-stage screening of PDCs in tauopathy models, primarily as toxicity studies, while also highlighting the need for further mechanistic and pharmacokinetic studies.

## Materials and methods

### Materials

*N*-acetyl cysteine (NAC), Okadaic acid, tetrazolium Bromide (MTT), cOmplete™ PhosSTOP™, mini protease inhibitor cocktail, and immobilon® -FL PVDF membrane were purchased from Sigma Aldrich (Dorset, UK). Polyacrylic acid (MW = 25,000 g/mol) was purchased from Fujifilm Wako Pure Chemical Corporation (Osaka, Japan). Tris(hydroxymethyl)aminomethane hydrochloride (Tris-HCl), dimethyl sulfoxide (DMSO), sodium chloride, triton X-100, sodium deoxycholate, sodium dodecyl sulphate, sodium orthovanadate, sodium fluoride, 2-mercaptoethanol, glycerol, bromophenol blue, Tris, glycine, methanol, Tween 20, bovine serum albumin, Pierce™ BCA Protein Assay Kit were purchased from Fisher Scientific (UK). 4–20% Mini-PROTEAN® TGX™ precast protein gels, 10-well, 50 µl, and precision plus protein dual colour standards were purchased from BIO-RAD (UK). Anti-Tau antibody (TAU-5, ab80579) and anti-GAPDH antibody (ab37168) were purchased from Abcam (UK). DMEM media, trypsin–EDTA (0.05%), Penicillin–Streptomycin (Pen/Strep, 10,000 U/mL), fetal bovine serum (FBS), MEM non-essential amino acids solution (NEAA), phospho-Tau (Ser202, Thr205) monoclonal antibody (AT8, MN1020), and phospho-Tau (Thr231) monoclonal antibody (AT180, MN1040) were purchased from ThermoFisher Scientific (UK). phospho-Tau (Ser396) antibody (PHF-13, sc-32275) was purchased from Santa Cruz Biotechnology. Intercept® (TBS) blocking buffer, IRDye® 680RD goat anti-rabbit IgG, and IRDye® 800CW goat anti-mouse IgG secondary antibody were purchased from LI-COR Biosciences (UK). The undifferentiated SH-SY5Y human neuroblastoma cell line was purchased from the European Collection of Authenticated Cell Cultures (ECACC, catalogue no. 94030304). Nutri-Fly® Bloomington Formulation was purchased from Scientific Laboratory Supplies (UK). *Drosophila* stocks were purchased from BDSC (https://bdsc.indiana.edu/).

### Methods

#### General cell culture technique

Human neuroblastoma cells were cultured according to the methods of Smith et al.^16^ with minor modifications. Briefly, undifferentiated SH-SY5Y cells were maintained in Dulbecco’s Modified Eagle Medium (DMEM) supplemented with 10% (v/v) FBS, 1% (v/v) NEAA, and 1% (v/v) Penicillin / Streptomycin. Cells were grown in a humidified incubator at 37 °C with 5% CO_2_. Upon reaching 80% confluence, cells were passaged, centrifuged, counted, and seeded into appropriate plates or flasks for further experiments.

#### Cell-uptake assay

Undifferentiated SH-SY5Y cells (5000 cells/100 µL/well) were seeded in a 10-well chamber slide (Greiner Cell View-543,079) and allowed to attach for 24 h at 37 °C with 5% CO₂. Subsequently, treatments were prepared in 10 mM Tris-HCl buffer (pH 7.4), further diluted in serum-free media, and added to the respective wells at the following concentrations: NM15 at 12.5 µg/mL, N5 at 44 µg/mL, and N5NM15 at a ratio of 3.5:1 (44:12.5 µg/mL). Tris-HCl buffer (10 mM, pH 7.4) was added as the solvent control. Live-cell imaging was performed using an AXR confocal microscope (Nikon, UK) with excitation 405 nm and emission 420–521 nm. Images were captured at 20 × magnification every 4 h for 24 h to monitor drug uptake.

#### Cell viability in the presence of okadaic acid using the MTT assay

Undifferentiated SH-SY5Y cells (10,000 cells/100 µL/well) were seeded into a 96-well plate and incubated for 24 h at 37 °C with 5% CO_2_ to allow cell attachment. OA (70 nM), was then added to all the wells except to solvent control wells, where serum-free DMEM was added. The plate was then incubated for an additional 24 h at 37 °C with 5% CO_2_. Subsequently, treatments were prepared in 10 mM Tris-HCl buffer (pH 7.4), further diluted in serum-free media, and added to the respective wells at the following concentrations: NAC at 12.5 µg/mL; PAA at 44 µg/mL; N5NM15 at a ratio of 3.5:1 (44:12.5 µg/mL). Tris-HCl buffer (10 mM, pH 7.4) was added to the solvent control wells. Following another 24 h incubation at 37 °C with 5% CO_2_, DMEM was removed, and 100 µL sterile-filtered (0.22 µm) MTT solution (1 mg/mL in serum-free DMEM) was added to each well, ensuring the procedure was performed in the dark. After the plates were then incubated for 4 h at 37°C with 5% CO_2_, the MTT solution was removed, and 200 µL DMSO was added to each well. The plates were shaken in the dark at room temperature for 15 min, and absorbance was measured at 595 nm (BioTek Synergy HT microplate reader, Agilent, UK). The percentage absorbance, representing cell viability, was calculated relative to the okadaic acid control alone (100% cell viability).

#### Western blot analysis of Tau protein expression in undifferentiated SH-SY5Y Cells

Undifferentiated SH-SY5Y cells were seeded at 2.5 × 10^6^ cells in cell culture (T75) flasks and allowed to attach for 48 h at 37 °C with 5% CO_2_. OA (20 nM), was added to all the flasks. Additionally, flasks where OA was not added, serum-free DMEM was added. The flasks were then incubated for an additional 24 h at 37°C with 5% CO_2_. Subsequently, treatments were prepared in 10 mM Tris-HCl buffer (pH 7.4), further diluted in serum-free media, and added to the respective flasks with and without okadaic acid at the following concentrations: NAC at 12.5 µg/mL; PAA at 44 µg/mL; N5NM15 at a ratio of 3.5:1 (44:12.5 µg/mL). Tris-HCl buffer (10 mM, pH 7.4) was added to the solvent control flasks. Following another 24 h incubation at 37 °C with 5% CO_2_, DMEM was removed and cells were lysed in a buffer containing 50 mM Tris-HCl (pH 8.0), 150 mM NaCl, 0.1% Triton-X-100, 0.1% SDS, 1 mM NaF, 0.5% C_24_H_39_O_4_Na, 1 mM Na_3_VO_4_, and one tablet of cOmplete™ PhosSTOP™ and mini protease inhibitor cocktail (Roche Diagnostics) per 10 ml buffer. Cell lysates were centrifuged at 15,000 × *g* for 20 min in a 4 °C precooled centrifuge (Heraeus multifuge 1 L-R, Heraeus, UK). The protein content of the supernatants was measured using a Pierce BCA Protein Assay kit. Protein (20 µg) was resolved on 4–20% Mini-PROTEAN® TGX™ precast protein gels for western blot analysis. The membranes were incubated overnight at 4 °C with primary antibodies Tau5, AT180, PHF-13 (1:1000), AT8 (1:500), and anti-GAPDH (1:5000) in Intercept® (TBS) blocking buffer containing 0.2% Tween 20/0.01% SDS solution. The membranes were then incubated for 1 h at room temperature with IRDye® 680RD goat anti-rabbit IgG, and IRDye® 800CW goat anti-mouse IgG secondary antibodies (1:5000) in Intercept® (TBS) blocking buffer with 0.2% Tween 20 / 0.01% SDS solution. Blots were developed using a LI-COR Odyssey Fc system (LI-COR Biotechnology, UK), and data analysed with Image Studio Version 5.2.

#### *Drosophila* stock and culture

*Drosophila melanogaster* expressing either the retinal photoreceptor-specific *GMR*-GAL4 (BDSC_8605), *GMR*-hTau^2N4R^ (BDSC_51361), and *GMR*-hTau^2N4R+GSK3β^ (BDSC_93134) and pan-neuronal driver *ELAV*-GAL4 (BDSC_458), UAS-Tau^2N4R^ (BDSC_51362), and Oregon-R (WT) (BDSC_2376) flies were obtained from the Bloomington Stock Centre (Indiana, USA). The flies were reared on Nutri-Fly® Bloomington Formulation, and the flies were maintained in an incubator at 23 °C.

The *ELAV*-GAL4 (BDSC_458) females were crossed to UAS-hTau^2N4R^ (BDSC_51362) and UAS-Oregon-R (WT) (BDSC_2376) male flies to generate *ELAV*/UAS-hTau^2N4R^ and *ELAV*/Oregon-R (WT) progeny, respectively.

#### Toxicity evaluation of N5NM15 nano-polyplex and PAA

The toxicity study was carried out as previously reported by Bernardes et al.^17^ with minor modifications. *GMR*-GAL4 and *ELAV*-GAL4 adult flies (0–3 days post-emergence) were tested (n = 18, six flies per tube in triplicate, 1:1 male-to-female ratio). Flies were reared on standard food (untreated control), food supplemented with 1.2 mM Tris-HCl buffer (pH 7.4, solvent control), and food supplemented with N5NM15 (low concentration (LC): 3.5:1 µg/mL, high concentration (HC): 44:12.5 µg/mL) and PAA (44 µg/mL). Flies were maintained at 23°C in an incubator, fed for 15 days, transferred every 7 days to new vials supplemented with the given treatments, and mortality was recorded daily. Progeny (0–3 days post-emergence) from supplemented vials underwent similar testing (n = 60, 20 flies per tube in triplicate, 1:1 male-to-female ratio). Maintained at 23 °C, they were fed for 15 days, transferred every 3–4 days to new vials supplemented with the given treatments, and mortality was recorded daily. Flies in the solvent control group were compared to standard food controls, while flies supplemented with N5NM15 and PAA were compared to the solvent control.

#### Assessment of rough eye phenotype in *Drosophila* model of Tauopathy using scanning electron microscopy (SEM)

*GMR*-GAL4, *GMR*-hTau^2N4R^ and *GMR*-hTau^2N4R+GSK3β^ adult flies were maintained at 23 °C and reared on the following types of media (A) standard (untreated control), (B) supplemented with 1.2 mM Tris-HCl buffer (pH 7.4, solvent control), (C) supplemented with N5NM15 (LC: 3.5:1 µg/mL, HC: 44:12.5 µg/mL) and (D) PAA (44 µg/mL). Parents were discarded, and F1 progeny from each *Drosophila* model were collected for 11 days and transferred every 3–4 days to new vials supplemented with respective treatments.

For SEM analysis, flies were euthanised and dehydrated for 90 min at increasing ethanol concentrations (40%, 50%, 60%, 70%, 80%, 90%, 96%, 99%, 100%)^18^. Following dehydration, fly samples were incubated overnight in hexamethyldisilazane, and dried under *vacuo*. Dried flies were mounted onto JEOL SEM stubs before sputter coating with gold for 4 min on a Quorum Q150R ES sputter coater and examined under a JEOL JSM-7100F LV FEG-SEM (JEOL, UK) at 180 × magnification and 10.0 kV for rough eye phenotype assessment^18^. Images were processed using ImageJ to measure eye length. Quantitative analysis of the total distance ommatidial disorderliness index of all stable ommatidia [Odld] was performed using the Flynotyper ImageJ plugin^19^. Ommatidia of 150 were considered and ranked by stability.

#### Negative geotaxis assay

The negative geotaxis assay was carried out as previously reported by Sealey et al.^20^ with minor modifications to evaluate locomotor function in *ELAV*-Tau^2N4R^ and *ELAV*-Oregon-R (WT) male progeny. Flies were reared on media described above (Sect. “Assessment of rough eye phenotype in drosophila model of tauopathy using scanning electron microscopy (SEM)”). Parental flies were discarded after laying eggs, and male progeny were collected over 11 days. The flies were maintained at 23 °C and transferred every 3–4 days to new vials supplemented with the given treatments. For the assay, 20–30 age-matched male flies per genotype were placed in a clean 15 cm plastic vial marked with an 8 cm horizontal line and acclimated for 30 min. Flies were gently tapped to the bottom and given 20 secs to climb as a negative geotaxis response. The number of flies that successfully climbed above the 8 cm mark was recorded. Climbing performance was recorded on three biological cohorts and repeated three times for each group with 30 secs break between each trial. The averaged data, represented as percentages, were calculated by dividing the number of flies above the 8 cm mark by the total number of flies tested within each group. The assay was performed weekly for three weeks to monitor changes in locomotor function over time.

#### Survival assay

The survival assay was performed to assess the lifespan of *ELAV*/UAS-Tau^2N4R^ and *ELAV*/Oregon-R (WT) male and female progeny, following the same treatment conditions as the negative geotaxis assay. Flies were reared on the following types of media (A) standard (untreated control), (B) supplemented with 1.2 mM Tris-HCl buffer (pH 7.4, solvent control), (C) supplemented with N5NM15 (LC: 3.5:1 µg/mL, HC: 44:12.5 µg/mL). Parental flies were discarded after egg-laying, and male and female progeny were collected over 11 days. Age-matched flies (20–30 per genotype) were placed in separate vials and transferred every 3–4 days to new vials with the respective treatments. The number of surviving flies was recorded weekly for 6 weeks.

### Statistical analysis

Results are presented as mean ± SEM from three independent experiments (n = 3) unless otherwise stated. Statistical analysis was performed using GraphPad Prism 10 (GraphPad Software, USA). Data normality was assessed using the Shapiro-Wilk test, and all datasets were confirmed to follow a normal distribution (*p* > 0.05). Significant differences between groups were determined using the one-way ANOVA followed by Dunnett’s multiple comparisons tests and Mantel-Cox log-rank test for survival data (*p < 0.05, **p < 0.01, ***p < 0.001, ****p < 0.0001, ns denotes not significant).

## Results and discussion

### Cell-uptake assay

Confocal microscopy was used to qualitatively assess the cellular uptake of NM15, N5, and N5NM15 nano-polyplexes in undifferentiated SH-SY5Y cells over 24 h (Fig. 1D, for grey-scale microscopy images, Figure S1). N5NM15 at a concentration of 44:12.5 µg/mL (mass ratio 3.5:1) and PAA at 44 µg/mL were used in all studies. These concentrations were selected based on prior in vitro viability assays conducted in undifferentiated SH-SY5Y cells^15^. At 0 h, minimal fluorescence was detected across all treatment groups, indicating minimal internalisation. However, after 24 h, an increase in fluorescence intensity was observed in NM15-treated cells, along with noticeable morphological changes, including disrupted cell structures. This observation aligns with previous viability studies^15^, which showed that NM15 reduced undifferentiated SH-SY5Y cell viability to approximately 60%, suggesting that NM15 induces cytotoxic effects. The fluorescence observed in NM15 might be attributed to the formation of a new conjugated system^21,22^, the imino bond (HC = N), in NM15 (Fig. 1A).

In contrast, N5 and N5NM15-treated cells exhibited visibly lower fluorescence compared to NM15 treated cells, and maintained their morphology, consistent with their higher cell viability (approximately 90%)^15^. The fluorescence in N5 can be attributed to the presence of the 1,8-naphthalimide moiety (Fig. 1B), which is known for its intrinsic fluorescence due to the conjugated π-π* system^23^. However, the N5NM15 nano-polyplex did not exhibit a substantial increase in fluorescence compared to NM15 alone. This suggests that a potential π-π* interaction between the aromatic rings of the 1,8-naphthalimide group and vanillin could lead to fluorescence quenching or a shift in the emission wavelength. Additionally, while the formation of compact complexes is expected, the flexibility of the linker chains and polymer backbones likely hinders the optimal orientation of the 1,8-naphthalimide group around vanillin^15^. This reduced efficiency of interaction could reduce the fluorescence property of N5NM15.

NM15 fluorescence appeared more widely distributed throughout the cells, whereas N5 and N5NM15 fluorescence was observed to be more localised in specific intracellular regions. PDCs are commonly internalised via endocytosis and trafficked to lysosomes^24,25^, suggesting that the observed localisation may reflect typical intracellular trafficking pathways. Such localisation is a fundamental mechanism for PDC nanoparticles at the cellular level, as lysosomotropic transport via endocytosis provides an optimal pathway for the release of bioactive agents not susceptible to proteolytic degradation^24,25^. It is important to note that NM15 is not a nanoparticle, whereas both N5 and N5NM15 exist as nanoparticles, which may contribute to the differences in cellular uptake and localisation. While our confocal data indicate successful internalisation of NM15, N5, and N5NM15 into undifferentiated SH-SY5Y cells, definitive conclusions about their precise intracellular trafficking require further investigation. Future studies incorporating co-localisation with organelle-specific markers (e.g., lysosomes, nuclei, cell membranes) will aim to provide clearer insights into these mechanisms.

### In Vitro cellular protective effects against okadaic acid-induced neurotoxicity and hyperphosphorylation of tau protein

Hyperphosphorylation of tau due to reduced activity of protein phosphatases 1 and 2A (PP1/PP2A) has been implicated in neurofibrillary tangle formation in AD^26^. Okadaic acid (OA), a potent and selective inhibitor of serine/threonine phosphatases 1 and 2A, is widely used as a stressor to induce tau hyperphosphorylation in vitro and *in vivo*^26^. Additionally, OA is a well-known oxidative stress inducer that contributes to neurotoxicity by promoting lipid peroxidation, depleting intracellular glutathione (GSH), and impairing antioxidant enzyme activity^26^.

**Fig. 2:**
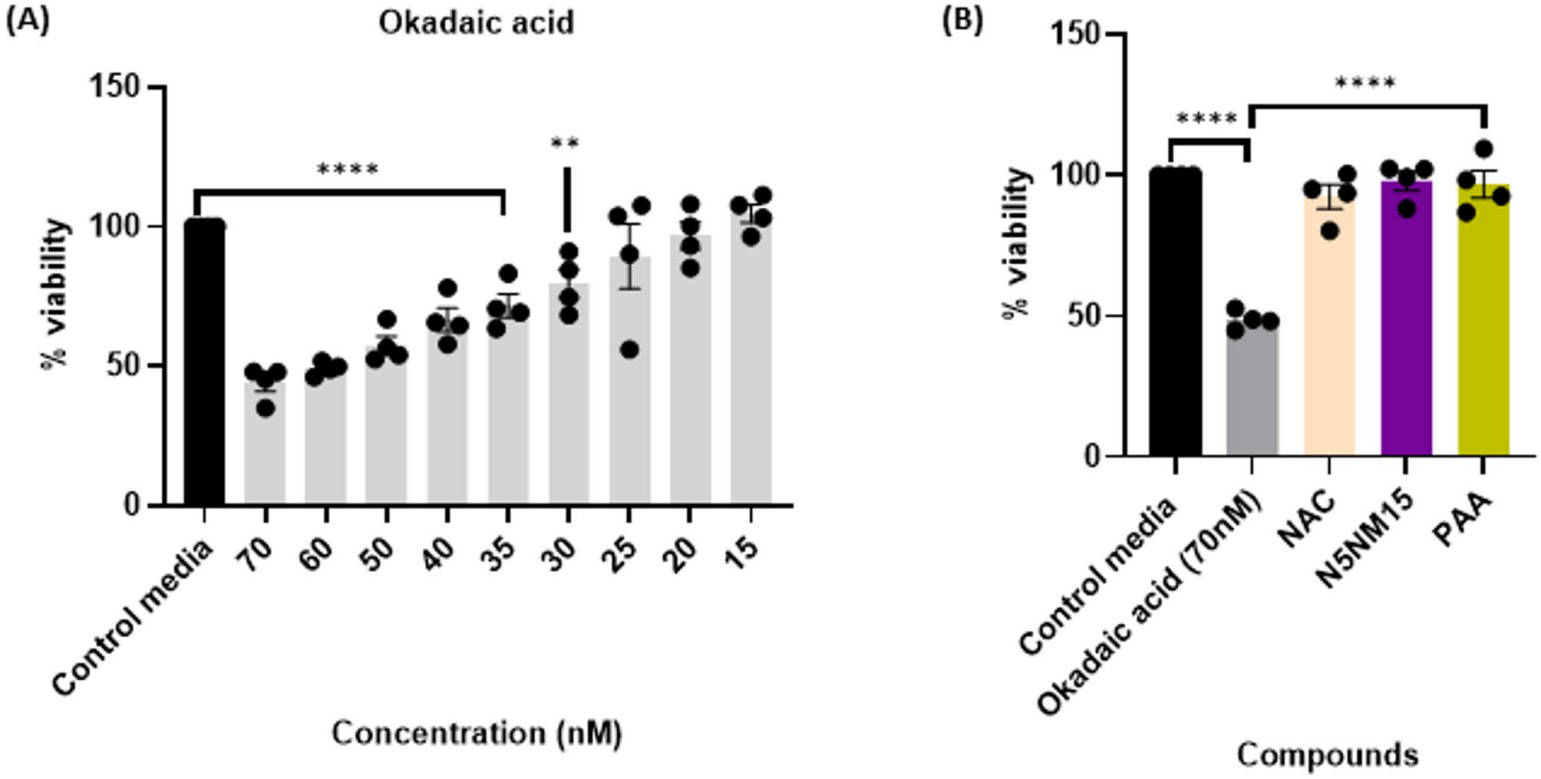
Illustrates (**A**) Cell viability of OA in undifferentiated SH-SY5Y cells determined using an MTT assay after 24 h. (**B**) Cellular protective effects of NAC, N5NM15, and PAA against OA-induced neurotoxicity in undifferentiated SH-SY5Y cells determined using an MTT assay after 24 h. Data presented as mean ± SEM (n = 4 independent experiments), significant difference via One-way ANOVA (****p ≤ 0.0001), and Dunnett’s multiple comparisons tests.

In this study, OA was used as a stressor to induce tau hyperphosphorylation in undifferentiated SH-SY5Y. The MTT assay was employed to determine the optimal concentration of OA for inducing neurotoxicity in undifferentiated SH-SY5Y cells (cell viability below 50% at 70 nM, Fig. 2A). Subsequently, the protective effects of the N5NM15 nano-polyplex and PAA against OA-induced neurotoxicity were assessed, with *N*-acetyl cysteine (NAC, concentration of 12.5 µg/mL) used as a positive control. Both N5NM15 and PAA significantly protected undifferentiated SH-SY5Y against OA-induced neurotoxicity, enhancing cell viability by over 45% (*p* ≤ 0.0001) (Fig. 2B). These results highlight the ability of both N5NM15 and PAA to mitigate OA-induced neurotoxicity.

**Fig. 3:**
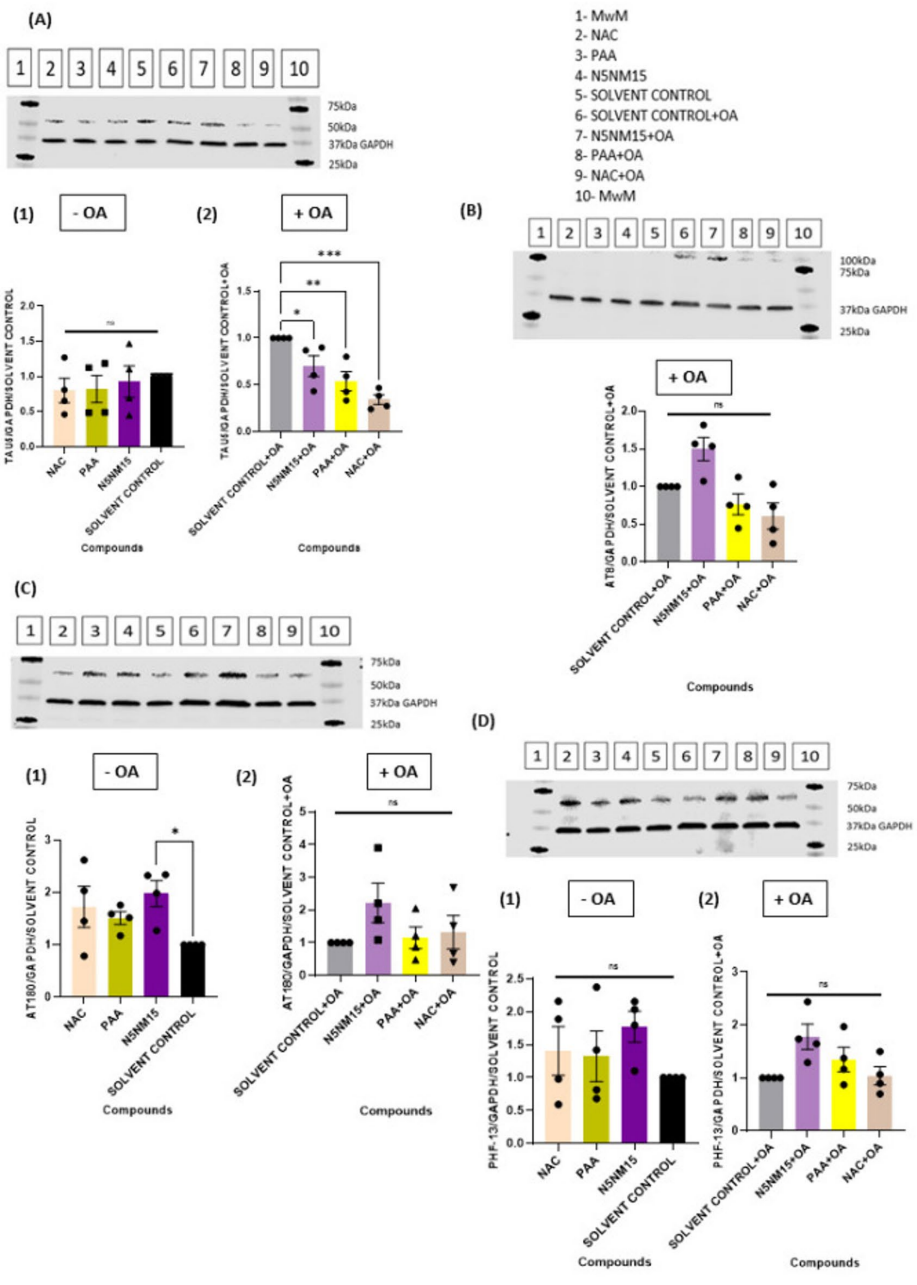
Illustrates the effects of compounds on various tau protein markers in undifferentiated SH-SY5Y cells under stress condition induced by OA. (**A**) Effect of compounds on total tau (Tau5) protein levels in undifferentiated SH-SY5Y cells stressed (1) without OA and (2) with OA (20 nM). (**B**) Effect of compounds on AT8 (pSer202/pThr205) protein levels in undifferentiated SH-SY5Y cells stressed with OA (20 nM). (**C**) Effect of compounds on AT180 (pThr231) protein levels in undifferentiated SH-SY5Y cells stressed (1) without OA and (2) with OA (20 nM). (**D**) Effect of compounds on PHF-13 (pSer396) protein levels in undifferentiated SH-SY5Y cells stressed (1) without OA and (2) with OA (20 nM). Data presented as mean ± SEM (n = 4), significant difference via One-way ANOVA (***p ≤ 0.001 for Figure A2, **p ≤ 0.01 for figure B, and *p ≤ 0.05 for Figure C1), and Dunnett’s multiple comparisons tests compared to solvent control/solvent control + OA.

The ability of these compounds to impact the levels of total and hyperphosphorylated tau levels in Undifferentiated SH-SY5Y cells was determined (Fig. 3). Undifferentiated SH-SY5Y cells express endogenous tau, predominantly the 0N3R isoform, though basal expression of 1N4R and 0N4R tau isoforms has also been detected in Undifferentiated SH-SY5Y cells^27^. To investigate the effects of N5NM15 and PAA on tau protein, Western blot analysis was performed using Tau5 (total tau, detecting both phosphorylated and non-phosphorylated tau), AT8 (pSer202/pThr205), AT180 (pThr231), and PHF-13 (pSer396) antibodies, respectively (Fig. 3).

The results revealed that neither N5NM15 nor PAA significantly reduced total tau protein levels in SH-SY5Y cells without okadaic acid (Fig. 3A1). This indicates that these compounds do not induce tau hyperphosphorylation under normal conditions, suggesting that they are non-toxic on their own. However, in OA-stressed SH-SY5Y cells, N5NM15 (1.43-fold) and PAA (1.89-fold) significantly reduced total tau levels (*p* ≤ 0.01) compared to the solvent control (Fig. 3A2). These results suggest that these compounds primarily modulate tau levels under pathological conditions rather than influencing basal tau expression. Interestingly, Chesser et al.^28^ reported that high concentrations of epigallocatechin-3-gallate (EGCG; 50 µM) reduced total tau levels, although this effect did not reach statistical significance. This aligns with the results and supports that specific polyphenols may modulate total tau levels under pathological conditions. N5NM15 has been shown to exhibit both antioxidant and cholinesterase activity^15^, which may contribute to its ability to modulate tau-related pathology by reducing oxidative stress and increasing cholinergic function, both of which are linked to tauopathies.

In contrast to total tau, neither N5NM15, PAA, nor NAC significantly reduced tau phosphorylation at the AT8, AT180, or PHF-13 sites, with or without OA (Fig. 3B–D). However, both NAC and PAA exhibited a reduction in AT8 expression (1.63 and 1.3-fold, respectively, Fig. 3B), suggesting a modest effect on tau phosphorylation at the pSer202/pThr205 site. The lack of significant changes at AT8, AT180, and PHF-13 sites suggests that these compounds do not broadly affect tau phosphorylation, at least at the tested concentrations. It is important to note that not all phosphorylated tau epitopes were studied, and the results are limited to a single concentration of NAC, N5NM15, and PAA. Therefore, the potential for a broader effect at different phosphorylation sites or at varying concentrations remains to be explored.

Although phosphorylated tau levels were significantly unaffected, the reduction in total tau levels under pathological conditions may still have therapeutic relevance. Previous studies indicate that lowering total tau can mitigate tau pathology and neurodegeneration, even without strong effects on specific phosphorylation epitopes^29,30^. For example, in the Tg(tau_P301L_)4510 repressible tauopathy mice model, suppression of tau transgene expression via doxycycline administration halted neuronal loss and restored memory function, despite the continued presence of neurofibrillary tangles^29^. This highlights that reducing total tau expression alone can rescue cognitive function and underscores the potential of total tau-lowering strategies. Overall, these findings suggest that N5NM15 and PAA reduce total tau under pathological conditions without broadly affecting phosphorylation, warranting further investigation into their therapeutic potential in AD and related tauopathies.

It is important to note that these experiments were conducted using undifferentiated SH-SY5Y cells, which have limitations as a neurodegenerative model due to their immature neuronal phenotype^31^. Future work should include more physiologically relevant systems, such as differentiated SH-SY5Y cells or human induced pluripotent stem cell (iPSC)-derived neurons^31,32^, to better assess the therapeutic potential and mechanisms of N5NM15 and PAA in neuronal contexts more closely resembling the human brain.

### In vivo toxicity and efficacy study of N5NM15 and PAA in *Drosophila* tauopathy models and age-matched controls

**Fig. 4:**
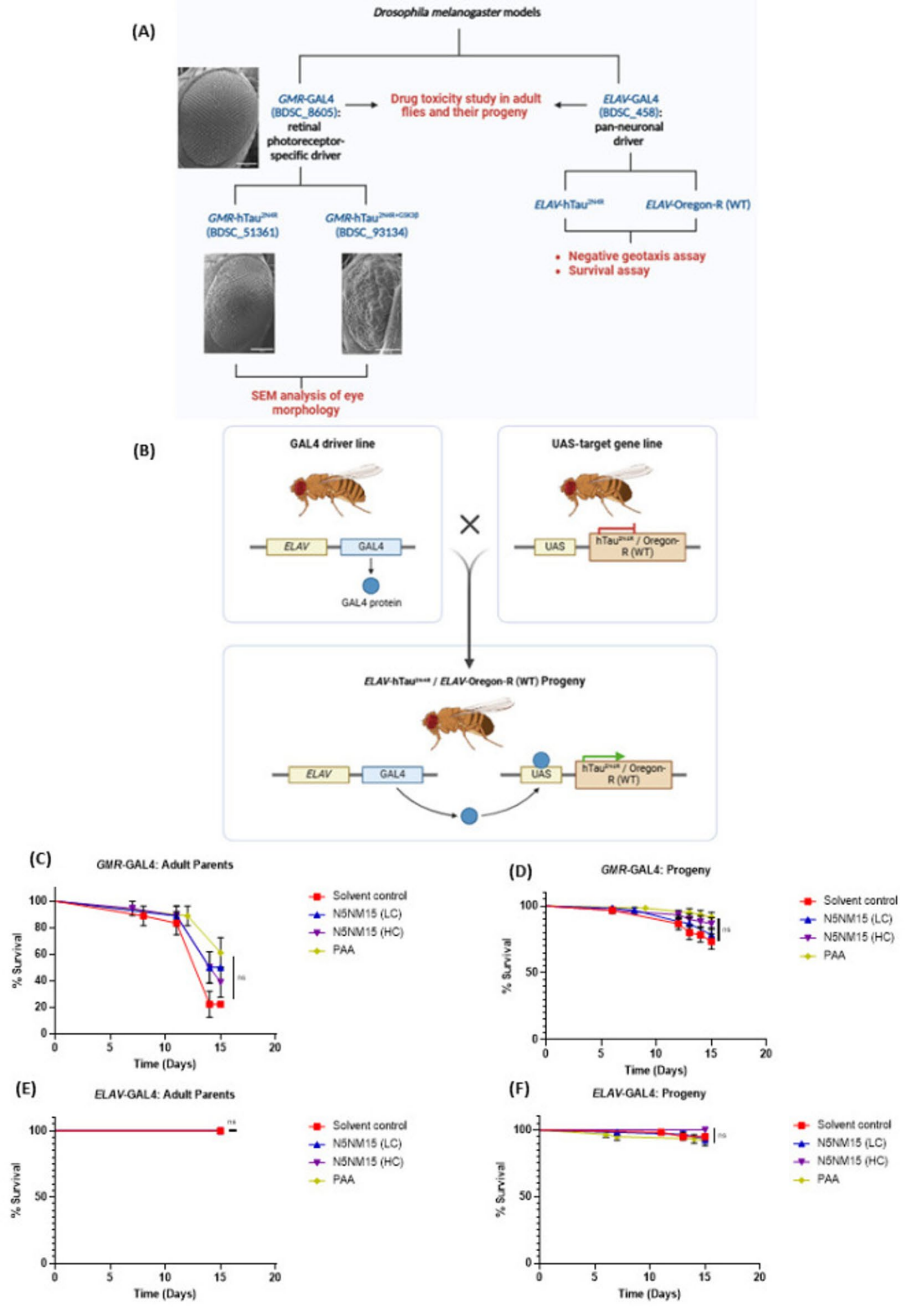
Evaluation of drug toxicity and tau pathology using *Drosophila melanogaster* models. Panel (**A**) showcases two experimental systems: the *GMR*-GAL4 driver (BDSC_8605), a retinal photoreceptor-specific, and the *ELAV*-GAL4 line (BDSC_458), a pan-neuronal driver. Tau pathology was studied on *GMR*-hTau^2N4R^ (BDSC_51361) and *GMR*-hTau^2N4R+GSK3β^ (BDSC_93134) progeny to assess retinal morphology using Scanning Electron Microscopy (SEM). Meanwhile, the *ELAV*-hTau^2N4R^ progeny was used for behavioral assays, including negative geotaxis (climbing ability) and survival assays, with *ELAV*-Oregon-R (wild type) flies serving as controls. Panel (**B**) explains the GAL4-UAS system, where virgin *ELAV*-GAL4 (BDSC_458) females are crossed with male UAS-hTau^2N4R^ (BDSC_51362) / UAS-Oregon-R (WT) (BDSC_2376) flies to produce pure *ELAV*-hTau^2N4R^ / *ELAV*-Oregon-R (WT) progeny. In these progenies, the GAL4 protein is expressed in neurons, activating UAS-hTau^2N4R^ to model Tau-induced neurodegeneration or serve as a wild-type control (*ELAV*-Oregon-R). Panel **(C-F),** toxicity study in *GMR*-GAL4 and *ELAV*-GAL4 adult flies and their progeny after feeding with the N5NM15 at varying concentrations (low concentration (LC) = 3.5:1 µg/mL, and a high concentration (HC) = 44:12.5 µg/mL) and PAA = 44 µg/mL. Statistical analysis was performed using the Mantel-Cox test, with n = 18 adult flies (6 flies per group in 3 separate tubes, maintaining a 1:1 male-to-female ratio) and n = 60 progeny (20 flies per group in 3 separate tubes, maintaining a 1:1 male-to-female ratio).

The in vivo toxicity and efficacy of N5NM15 and PAA were evaluated in *Drosophila* tauopathy models using two different GAL4 drivers: *GMR*-GAL4 (retinal photoreceptor-specific)^18^ and *ELAV*-GAL4 (pan-neuronal)^33^ (Fig. 4A and B).

To evaluate dose-dependent effects and further assess the safety of the nano-polyplex, two concentrations of N5NM15 were tested: a high concentration of 44:12.5 µg/mL (denoted as HC) and a substantially lower dose, representing a 12.5-fold reduction of each component (i.e., 3.5:1 µg/mL), referred to as the low concentration (LC). This dosing strategy enabled the investigation of both potential therapeutic effects and long-term tolerability of N5NM15 across a broad concentration range in wild-type and tauopathy *Drosophila* models.

The toxicity studies on *GMR*-GAL4 and *ELAV*-GAL4 revealed that N5NM15 at both low (3.5:1 µg/mL) and high (44:12.5 µg/mL) concentrations, as well as PAA (44µg/mL), were toxic to *GMR*-GAL4 adult parent flies after 10 days (Fig. 4C). For a comparison of the toxicity study on solvent control versus normal food, see Figure S2. However, toxicity was reduced in *GMR*-GAL4 F1 progeny, indicating a possible developmental adaptation (Fig. 4D). In contrast, no toxicity was observed at either concentration of N5NM15 and PAA in *ELAV*-GAL4 adult parents or progeny flies (Fig. 4E and F), suggesting that the treatment was well tolerated in the pan-neuronal model.

**Fig. 5:**
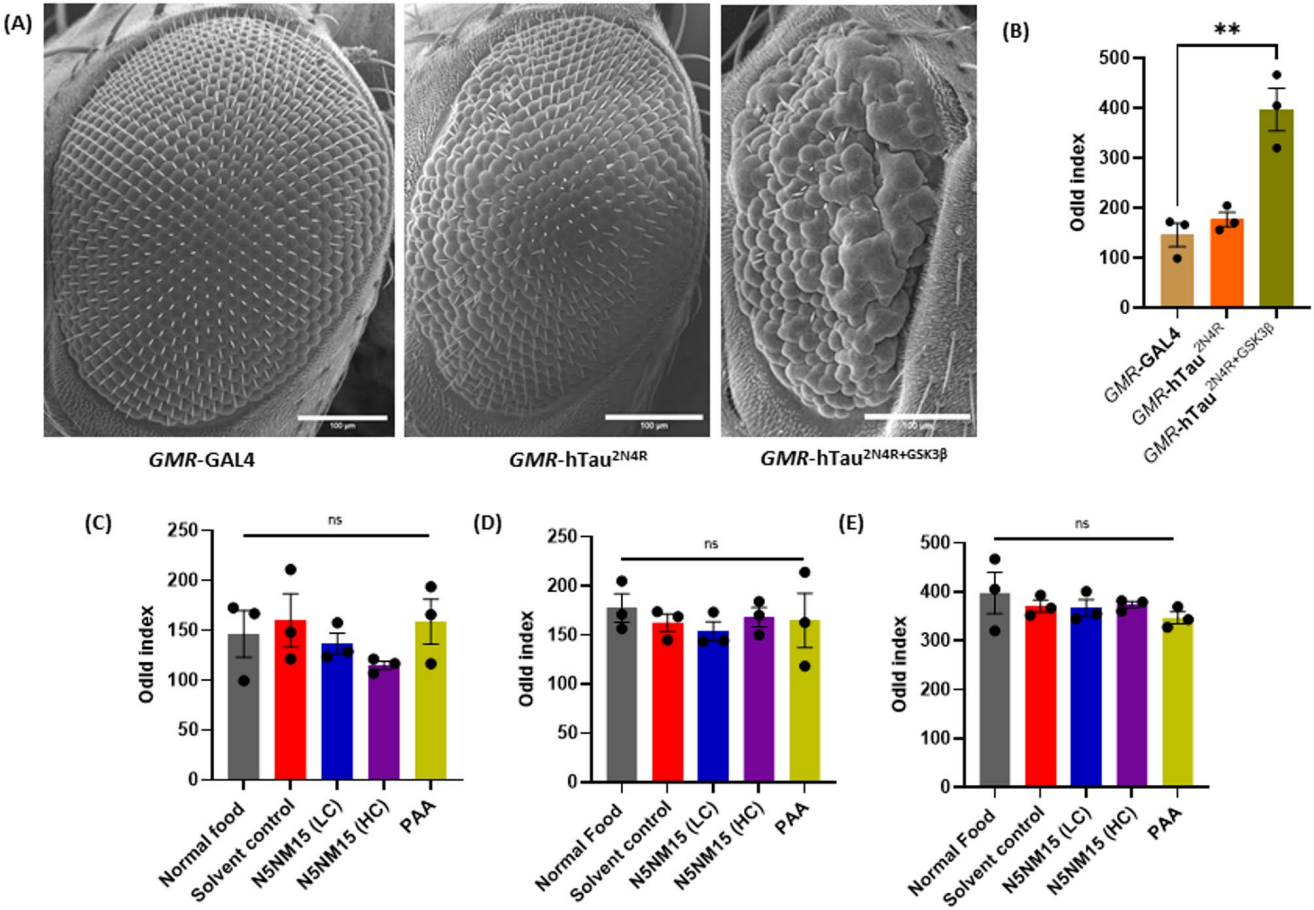
Analysis of rough eye phenotype (REP) in *GMR*-GAL4, *GMR*-hTau^2N4R^, and *GMR*-hTau^2N4R+GSK3β^ *Drosophila* models. (**A**) SEM images illustrating the difference in eye morphology between *GMR*-GAL4, *GMR*- hTau^2N4R^, and *GMR*- hTau^2N4R+GSK3β^ flies and (**B**) phenotypic score quantifying the REP based on disorderliness in the ommatidial arrangement (Odld- total distance ommatidial disorderliness index of all stable ommatidia). Phenotypic score quantifying the REP based on disorderliness in the ommatidial arrangement of (**C**) *GMR*-GAL4, (**D**) *GMR*-hTau^2N4R^, and (**E**) *GMR*- hTau^2N4R+GSK3β^ flies treated with LC and HC of N5NM15 and PAA. Data are presented as mean ± SEM (n = 3). Statistical analysis was performed using one-way ANOVA (**p ≤ 0.01 for Figure B), with Dunnett’s multiple comparisons test. Scale bar = 100 µm.

Furthermore, to assess the therapeutic potential of N5NM15 and PAA in tauopathy, phenotypic analysis was first conducted on *GMR*-GAL4 to observe any toxicity in the eyes using scanning electron microscopy (SEM). No toxicity was observed in the eyes of N5NM15 and PAA-treated flies, suggesting that the treatments did not induce any significant adverse effects on eye morphology (Figs. S3 and S4). Following this, analysis was extended to the *GMR*-hTau^2N4R^ and *GMR*-hTau^2N4R+GSK3β^ *Drosophila* models. *GMR*-hTau^2N4R^ expresses the 2N4R isoform of human tau (Microtubule-Associated Protein Tau (MAPT)) in the eye under the control of *GMR*, which leads to the accumulation of misfolded tau protein. This misfolding of tau promotes tau aggregation, resulting in a mild rough-eye phenotype^18^ (Fig. 5A and B). On the other hand, *GMR*-hTau^2N4R+GSK3β^, which additionally expresses Glycogen Synthase Kinase 3 Beta (GSK3β) kinase along with 2N4R under the control of *GMR*, exhibits markedly reduced eyes with abnormally fused ommatidia and complete loss of bristles^18^ (Fig. 5A and B). GSK-3β is a proline-directed serine/threonine kinase and one of the primary kinases responsible for the phosphorylation of tau. This hyperphosphorylation of tau leads to the formation of tau aggregates, which are a key feature of NDs such as AD^18^.

When treated with N5NM15 and PAA, there was no improvement in the disorderliness in the ommatidial arrangement of the fly’s eyes, nor was there a significant increase in eye bristles (Fig. 5C–E). Additionally, no significant change in eye length was observed following treatment (Figs. S3 and S4). These findings suggest that the N5NM15 nano-polyplex and PAA may not directly influence tau aggregation or phosphorylation, which primarily contribute to neurotoxicity in these models. Instead, their mechanism of action might be linked to alternative pathways, such as reducing oxidative stress or modulating inflammatory responses. In contrast, Sundararajan et al.^34^ administered cerium oxide nanoparticles to *GMR*-htau flies, which resulted in phenotypic rescue correlated with a reduction in htau mRNA expression and an upregulation of autophagy-related genes (ATG1 and ATG18). This study suggested that the enhancement of autophagic clearance of misfolded tau contributed to neuroprotection. This mechanistic clarity highlights a crucial limitation in the current study: while N5NM15 did not rescue external phenotypes, further investigation is required to explore whether it may influence intracellular mechanisms, such as oxidative stress responses or autophagy, which may not immediately translate to phenotypic rescue. Hence, future studies to more comprehensively understand the biological activity of the nano-polyplex should include qRT-PCR and Western blot analysis to assess changes in tau and autophagy pathway markers at both transcriptional and translational levels. These findings highlight that the efficacy of the N5NM15 nano-polyplex and PAA in tau-induced *Drosophila* degeneration appears limited.

### Effects of N5NM15 on locomotor function using negative geotaxis assay

Prior to assessing locomotor function, the effect of N5NM15 on progeny production was evaluated by determining the total number of F1 progeny flies collected after 11 days in both *ELAV*-Oregon-R (wild-type) and *ELAV*-hTau^2N4R^ flies. The results indicated no significant changes in progeny production, suggesting that N5NM15 does not adversely affect fertility or early developmental processes in *Drosophila* models (Fig. S5).

**Fig. 6:**
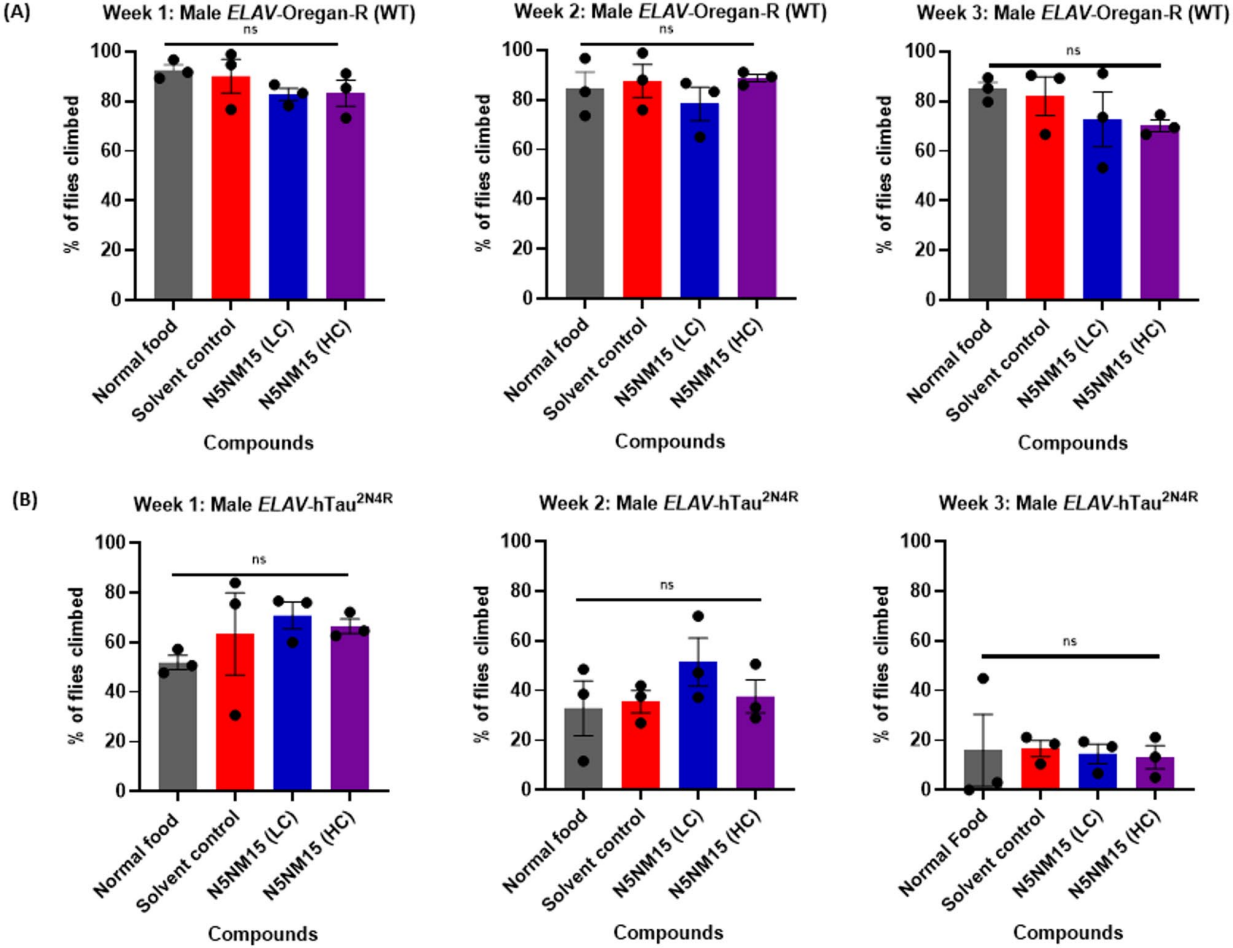
Negative geotaxis assay assessing climbing ability in (**A**) male *ELAV*-Oregon R (WT) flies and (**B**) male *ELAV*-hTau^2N4R^ flies for three weeks. Flies were treated with LC and HC N5NM15 and solvent control. Data are presented as mean ± SEM, with n = 75–80 male flies (20–30 flies per group, tested in three separate tubes). Statistical analysis was performed using one-way ANOVA.

To further evaluate the impact of N5NM15 on locomotor function, a negative geotaxis assay (climbing assay) (Video S6) was first conducted in male *ELAV*-Oregan-R (wild-type) flies to assess the general effect of N5NM15 in healthy flies before testing its efficacy in tauopathy models (Fig. 6A). Treatment with N5NM15 at both LC and HC, as well as the solvent control, did not significantly impact the climbing ability of the flies (above 80%) over the three weeks. However, a slight decline in climbing ability (approximately 70%) was observed in HC N5NM15-treated flies in the third week, suggesting a potential mild toxicity with prolonged exposure at higher concentrations. These findings indicate that N5NM15 is well-tolerated at lower doses in healthy flies, but prolonged exposure to higher concentrations may induce subtle locomotor impairments.

Subsequently, the climbing assay was performed in male *ELAV*-hTau^2N4R^ flies, a tauopathy model characterised by progressive locomotor deficits^19^ (Fig. 6B). In these flies, N5NM15 treatment at both LC and HC did not significantly improve climbing ability over a period of three weeks. However, during the first two weeks, flies treated with LC N5NM15 exhibited better climbing performance compared to those treated with HC N5NM15, the solvent control, and flies reared on normal food. These findings suggest that LC N5NM15 may exert a protective effect in the early stages of tauopathy, but this effect appears to diminish as the disease progresses. The relatively poorer climbing performance in HC N5NM15-treated flies compared to LC-treated flies indicates a potential dose-dependent effect, where higher concentrations could induce toxicity or cellular stress, ultimately reducing the therapeutic potential. However, findings from LC N5NM15 should be interpreted as preliminary and exploratory rather than definitive evidence of protective effects.

Importantly, Sheik Mohideen et al.^35^ demonstrated that the levels of sarkosyl-insoluble tau in neurons upon htau expression directly influence the climbing ability of *ELAV*-htau flies, and that improvements in locomotor function are associated with a reduction in insoluble tau species. Therefore, the lack of significant improvement in climbing ability in N5NM15-treated *ELAV*-hTau^2N4R^ flies could be attributed to the absence of changes in the levels of sarkosyl-insoluble tau. This highlights the need for future studies to examine whether N5NM15 affects tau aggregation or clearance at the biochemical level, particularly in relation to insoluble tau species.

### Survival assay of *Drosophila* models and long-term toxicity of N5NM15

**Fig. 7:**
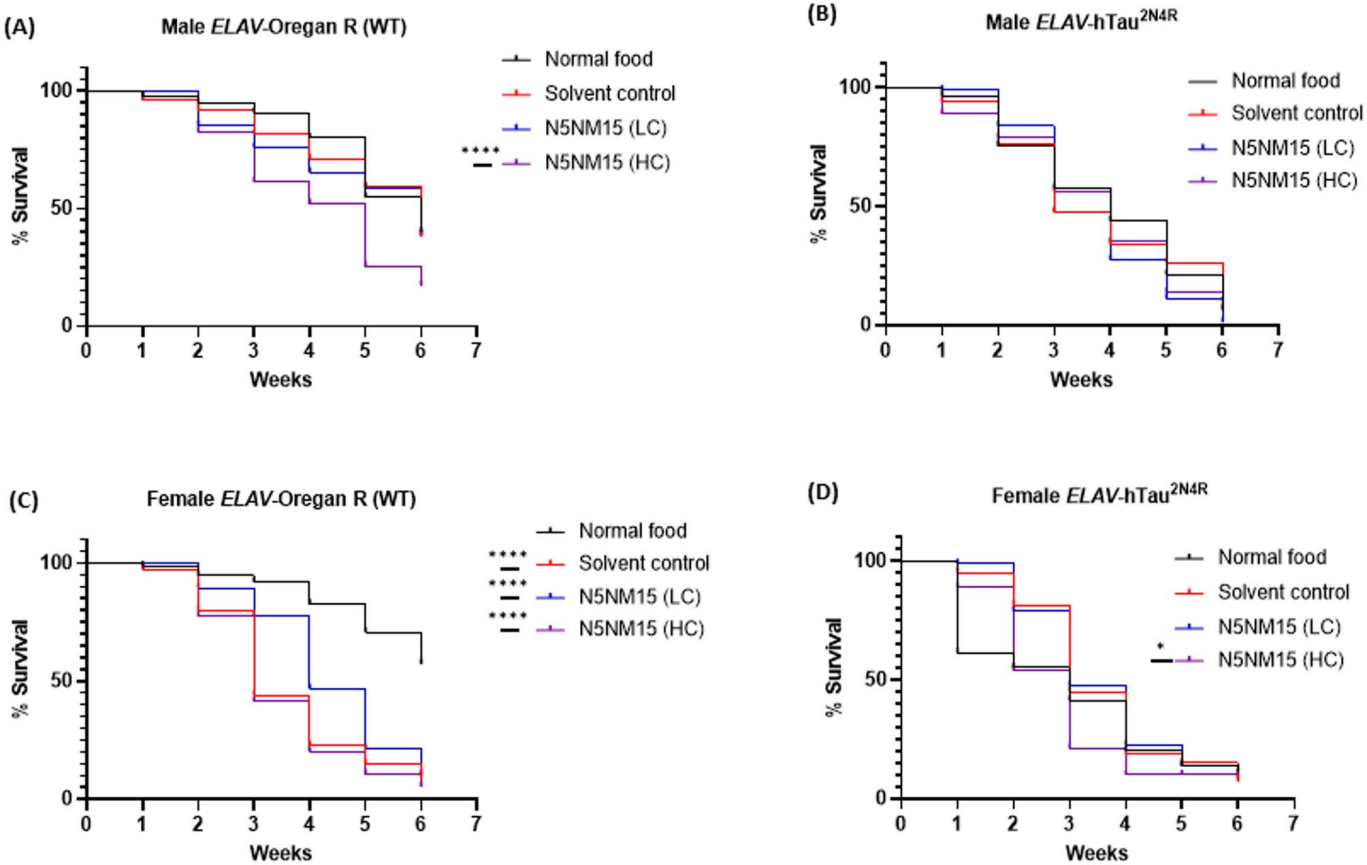
Survival assay for (**A** and **C**) male and female *ELAV*-Oregon R (WT) flies and (**B** and **D**) male and female *ELAV*-hTau^2N4R^ flies for six weeks. Flies were treated with LC and HC N5NM15, and solvent control. Statistical analysis was performed using the Mantel-Cox test, with n = 75–80 flies (75–80 male and 75–80 female flies, 20–30 flies per group, tested in three separate tubes for each sex).

Nanoparticles, including polymeric nanoparticles, have been shown to induce toxicity in *Drosophila* models, often manifesting as reduced survival and altered physiological responses, highlighting the need for careful evaluation of their long-term effects^36–38^. To evaluate the long-term toxicity of N5NM15, a survival assay was conducted in male and female *ELAV*-Oregon R (wild-type) flies over six weeks at LC and HC, along with a solvent control (Fig. 7A and C). In wild-type males, survival remained relatively stable in the normal food, solvent control, and LC-treated groups, indicating that LC N5NM15 did not exert significant long-term toxicity. However, flies treated with HC N5NM15 exhibited a significant decline in survival (****p ≤ 0.0001), suggesting that prolonged exposure to higher concentrations may induce toxicity over time. This effect was even more pronounced in wild-type females, where mortality was significantly higher in the solvent control, LC, and HC groups compared to the normal food group (****p ≤ 0.0001). The increased mortality in female flies across all treatment groups suggests that females may be more sensitive to both the solvent and N5NM15, possibly due to differences in physiological stress responses^39^. Further biochemical assays to assess drug toxicity, such as reactive oxygen species (ROS) assays and drug uptake studies, should be conducted to determine whether N5NM15 accumulates in specific tissues and contributes to toxicity through oxidative stress.

Subsequently, a survival assay was performed on *ELAV*-hTau^2N4R^ male and female flies (Fig. 7B and D). In tauopathy, the survival of flies is typically reduced after 3 weeks due to tau-induced neuronal dysfunctions^19^. During the first two weeks, *ELAV*-hTau^2N4R^ male and female flies treated with LC N5NM15 exhibited slightly improved survival compared to the normal food, solvent control, and HC N5NM15 groups. This suggests that LC N5NM15 may provide a potential early-stage protective effect in the tauopathy model. However, this benefit diminished over time, and by the later weeks, survival rates in the LC-treated group were comparable to the other groups. Moreover, while no significant improvement in survival was observed with these treatments, the HC N5NM15 group exhibited a significant reduction in survival in female flies (*p ≤ 0.05), which aligns with the results observed in the survival of wild-type females (Fig. 7C), where mortality was significantly higher.

While the survival assay results indicate a mild improvement in the early stages of development with low concentration N5NM15 treatment, these effects did not reach statistical significance and diminished over time. Similar to the climbing assay, these findings should be interpreted as preliminary and exploratory rather than definitive evidence of protective effects. Further biochemical assays are needed to clarify the underlying mechanisms and provide evidence supporting the protective effects of N5NM15.

An important factor to consider is the mode of compound administration. In this study, N5NM15 and PAA were delivered to flies via food supplementation. However, this method presents limitations in dosage control, as individual flies may consume varying amounts, leading to inconsistencies in drug exposure across groups. Moreover, in the absence of drug uptake and biodistribution studies, it remains unclear how much reaches the brain. These factors may have contributed to the variability and limited therapeutic effects observed. Future studies should consider more controlled delivery methods, such as microinjection, to ensure consistent dosing and perform drug uptake and localisation studies (e.g., fluorescence imaging or mass spectrometry) to confirm CNS delivery and improve the translational relevance of findings. Additionally, given that the solvent used in this study has shown long-term toxicity, it is worth considering a reformulation of the N5NM15 nano-polyplex. This reformulation could involve dissolving the nano-polyplex in a much lower concentration of Tris-HCl or another non-toxic buffer, which would help minimise any adverse effects while maintaining the therapeutic potential of the compound. Furthermore, the toxicity observed at higher concentrations underscores the need for further testing at lower doses and in different *Drosophila* models, where oxidative stress and inflammation can be studied to better understand its therapeutic potential.

Although *Drosophila melanogaster* is a widely used model for NDs research, its utility for evaluating high molecular weight polymers as well as polymer-drug nano-polyplexes may be limited. The structural and physiological differences between flies and mammals may affect the absorption, systemic distribution, and metabolism of such complex formulations. Consequently, validation in mammalian models, particularly rodents, would provide a more clinically relevant platform to assess brain-targeting efficiency, pharmacokinetics, and therapeutic outcomes of N5NM15 and similar nano-formulations.

## Conclusion

This study evaluates the toxicity profile of a novel nano-polyplex, N5NM15, and PAA in *Drosophila* models of tauopathy and undifferentiated human SH-SY5Y cells. The N5NM15 nano-polyplex demonstrated successful cellular uptake in SH-SY5Y cells, and both N5NM15 and PAA reduced total tau levels under pathological conditions without broadly affecting tau phosphorylation. While N5NM15 exhibited moderate toxicity in *GMR*-GAL4 adult flies, it was well-tolerated in *ELAV*-GAL4 flies, suggesting differential effects in *Drosophila* models. Despite promising results in cell culture, N5NM15 and PAA treatment did not improve tau-induced phenotypes or locomotor deficits in *Drosophila* tauopathy models. Climbing and survival assays revealed that lower concentrations of N5NM15 might exert mild protective effects in early tauopathy stages, but higher concentrations induced toxicity, impairing both locomotion and survival, particularly in female flies. These findings highlight the importance of optimising concentration and treatment duration for potential therapeutic applications, particularly in tau-related NDs. Further investigations are required to explore alternative mechanisms of action, such as modulating oxidative stress or inflammation, and to refine dosing strategies for improved efficacy. Reformulation of the polyplex using a much lower concentration of Tris-HCl or another non-toxic buffer may help minimise adverse effects while maintaining its therapeutic potential.

## Supplementary Information

Below is the link to the electronic supplementary material.

Supplementary Material 1

## Data Availability

All data supporting the findings of this study are provided within the manuscript and its Supplementary Information. No additional datasets were generated or analysed beyond those presented.
