# Supplementary material for: Evaluation of the toxicity and efficacy of a multi-target polymer-drug nano-polyplex in SH-SY5Y cells and Drosophila model of tauopathy
Source: Sci Rep. 2025 Nov 11;15:39454. doi: 10.1038/s41598-025-22924-0 (PMC12606271; doi:10.1038/s41598-025-22924-0)

**Supporting Information**

**Evaluation of the Toxicity and Efficacy of a Multi-Target Polymer-Drug Nano-Polyplex in Undifferentiated SH-SY5Y Cells and *Drosophila* Model of Tauopathy**

Nuruddin Mahadik^1^, Sri Nithya Paruchuri^2^, Rohina Arif^2^, Amanda S. Coutts^2^, Gemma A. Barron^1^, Paul Kong Thoo Lin^1^, Shreyasi Chatterjee^2*^, Colin J. Thompson^1*^

***^1^School of Pharmacy, Applied Sciences and Public Health, Robert Gordon University, Aberdeen, UK, AB10 7GJ***

***^2^Department of Science and Technology, Nottingham Trent University, Nottingham, UK, NG11 8NS***

^*^Corresponding authors

Colin J. Thompson ([c.thompson@rgu.ac.uk](mailto:c.thompson@rgu.ac.uk))

Shreyasi Chatterjee (shreyasi.chatterjee@ntu.ac.uk)


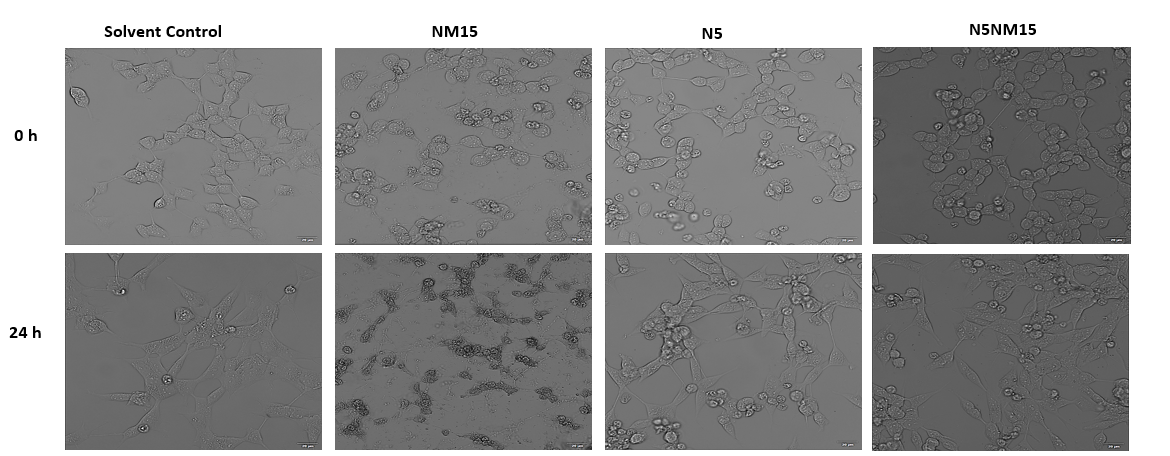


**Figure S1**. Grey-scale microscopy image depicting cellular uptake of NM15, N5 and N5NM15 nano-polyplex by undifferentiated SH-SY5Y cells incubated in serum-free DMEM media at 37^◦^C, with 5% CO_2_, images taken after 0 h and 24 h (Scale bar = 20 µm).


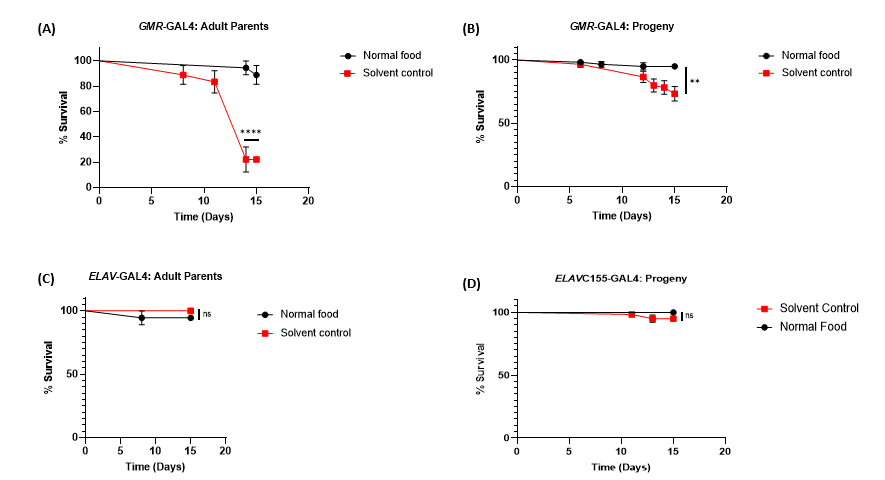


**Figure S2.** Toxicity study was assessed in *GMR*-GAL4 and *ELAV*-GAL4 adult flies and their progeny after feeding with the solvent control (1.2 mM Tris-HCl, pH 7.4) (**A-D**). Statistical analysis was performed using the Mantel-Cox test, with n=18 adult flies (6 flies per group in 3 separate tubes, maintaining a 1:1 male-to-female ratio) and n=60 progeny (20 flies per group in 3 separate tubes, maintaining a 1:1 male-to-female ratio).


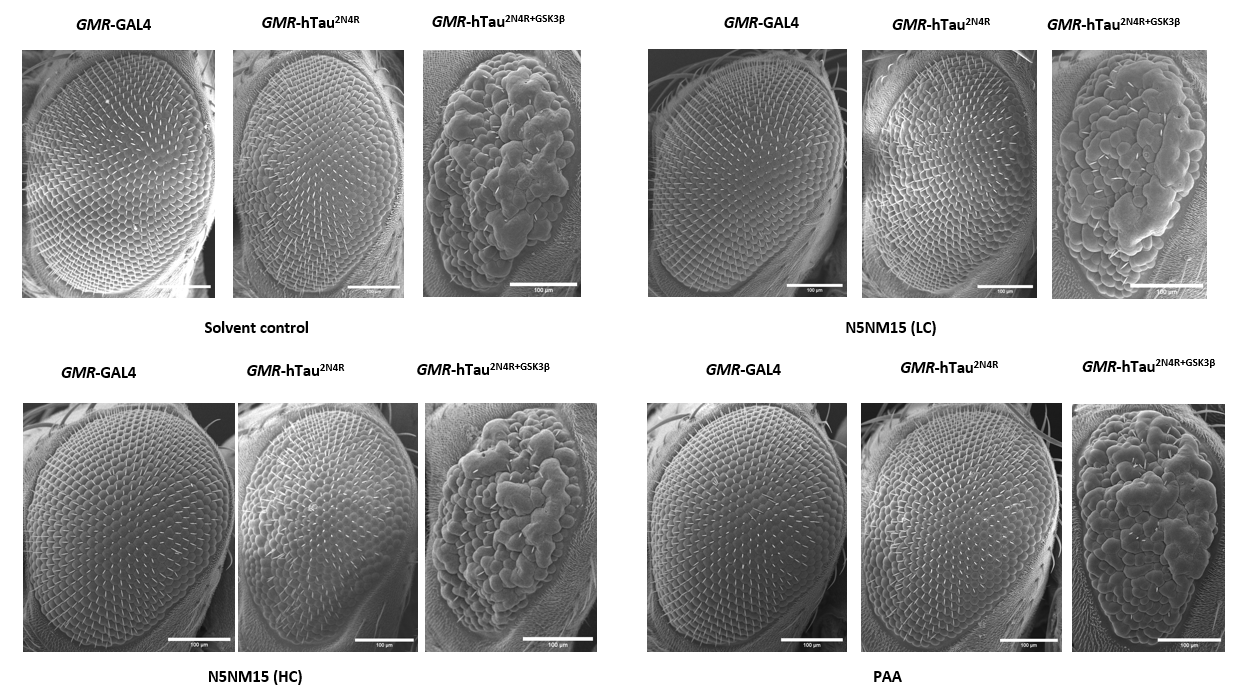


**Figure S3.** SEM images of *GMR-GAL4, GMR*-hTau^2N4R^ and *GMR*-hTau^2N4R+GSK3β^ flies treated with solvent control, LC and HC of N5NM15, and PAA illustrating their effects on eye morphology (Scale bar = 100 µm).


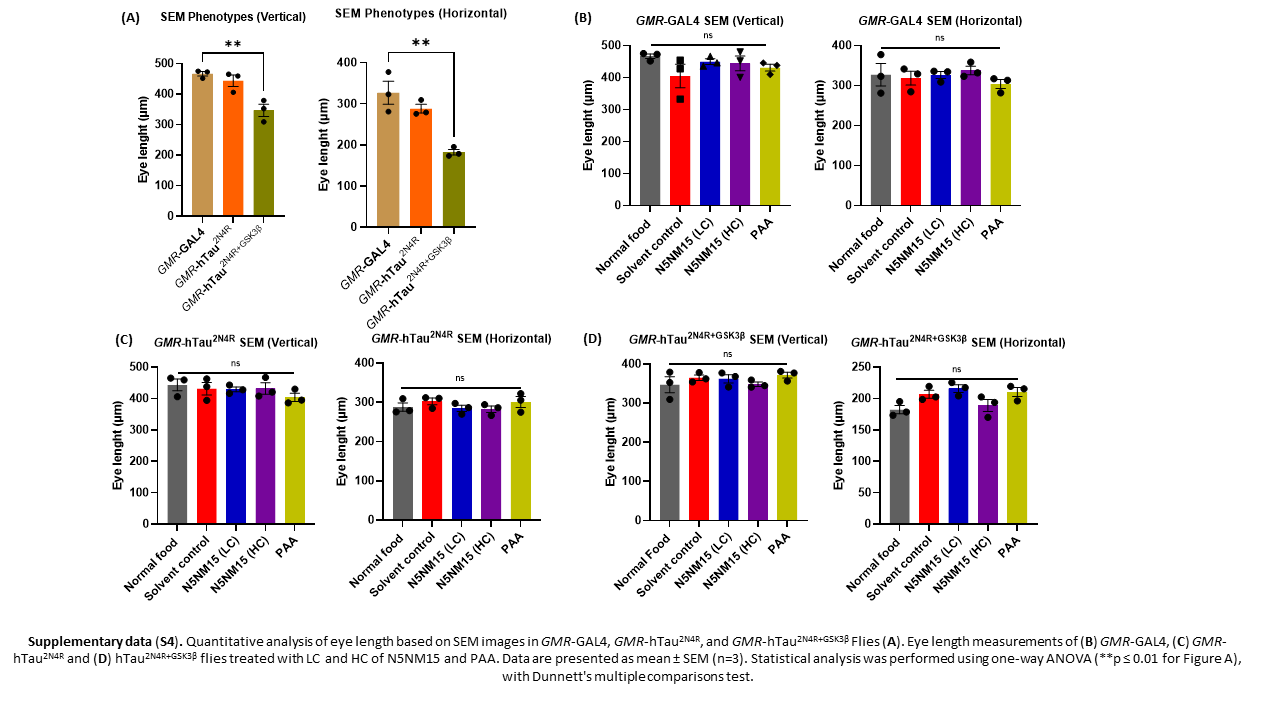


**Figure S4.** Quantitative analysis of eye length based on SEM images in *GMR*-GAL4, *GMR*-hTau^2N4R^, and *GMR*-hTau^2N4R+GSK3β^ Flies (**A**). Eye length measurements of (**B**) *GMR*-GAL4, (**C**) *GMR*-hTau^2N4R^ and (**D**) hTau^2N4R+GSK3β^ flies treated with LC and HC of N5NM15 and PAA. Data are presented as mean ± SEM (n=3). Statistical analysis was performed using one-way ANOVA (**p ≤ 0.01 for Figure A), with Dunnett's multiple comparisons test.


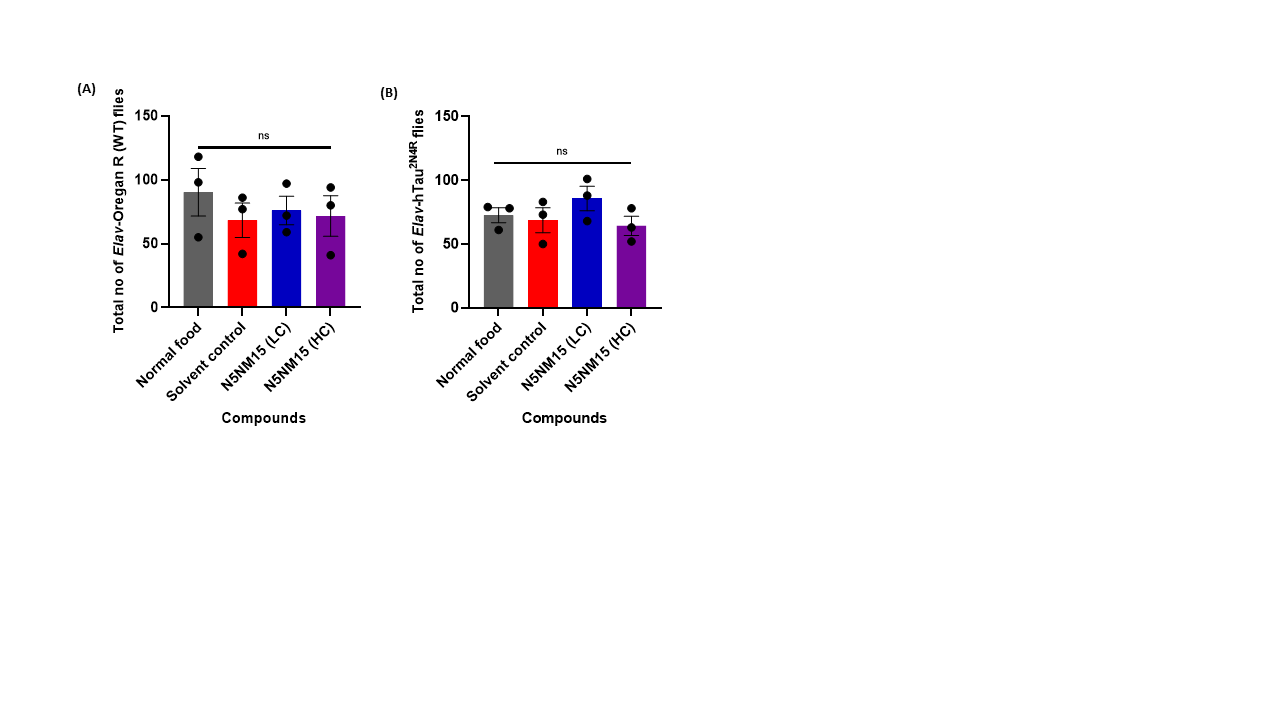


**Figure S5.** Total number of F1 progeny flies collected after 11 days to evaluate the effect of N5NM15 at varying concentrations (LC (11:3 µg/mL), and HC (44:12.5 µg/mL)) on progeny production. (**A**) *Elav*-Oregon-R (WT) flies were reared on normal food, solvent control, or food containing LC and HC of N5NM15. (**B**) *Elav*-hTau^2N4R^ progeny flies, expressing human tau protein, were reared under the same conditions. Data are presented as mean ± SEM (n=3). No significant differences (p ˃ 0.05, one-way ANOVA) were observed.

**Video S6.** Negative geotaxis assay: *ELAV*-hTau^2N4R^ vs *ELAV*-Oregon-R (WT) progeny flies in normal food.

**Full Blot images**

**1. Tau5- Figure 3A in the manuscript**


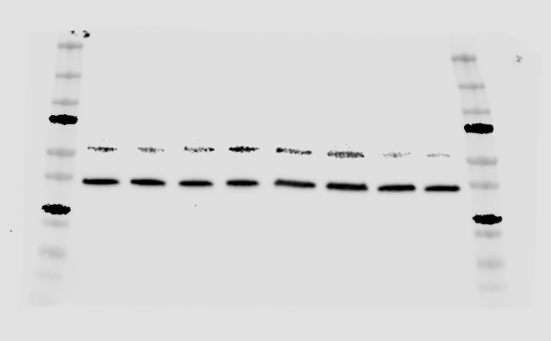


**2. AT8- Figure 3B in the manuscript**


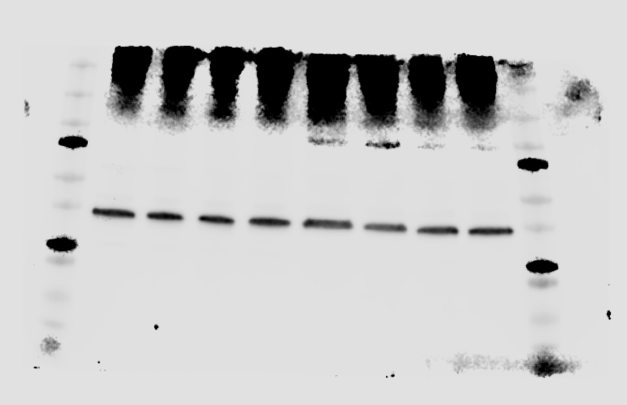


**3. AT180- Figure 3C in the manuscript**


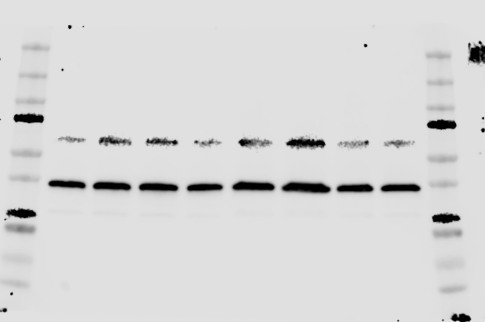


**4. PHF-13- Figure 3D in the manuscript**


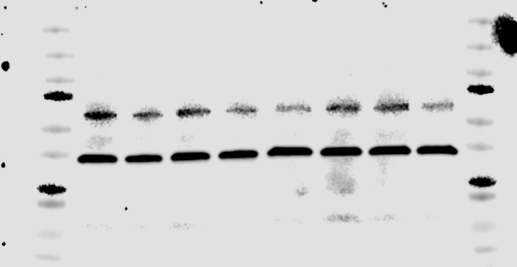

Supplement: Supplementary file 1 — Supplementary Material 1 [file 41598_2025_22924_MOESM1_ESM.docx]
